# Ollier's Disease of the Iliac Bone with Sacroiliac Joint Involvement in an Adolescent Patient

**DOI:** 10.1155/2016/4893718

**Published:** 2016-11-13

**Authors:** Olga D. Savvidou, George D. Chloros, Panagiotis Koutsouradis, Evangelia Skarpidi, Panayiotis J. Papagelopoulos

**Affiliations:** ^1^First Department of Orthopaedic Surgery, University of Athens School of Medicine, Attikon Hospital, Athens, Greece; ^2^417 VA Hospital (NIMTS), Athens, Greece; ^3^Hygeia Hospital, Athens, Greece

## Abstract

Ollier's disease of the hip bone involving the sacroiliac joint has not yet been reported in the English-language literature in both the mature and immature skeletons. The authors present such a unique case in an adolescent girl that posed a significant diagnostic challenge secondary to the rarity of the lesion and atypical clinical picture.

## 1. Introduction

Ollier's disease is a rare disease consisting of multiple enchondromas, usually metaphyseal, with the most frequent location being the metacarpals, metatarsals, and phalanges of the fingers and toes [1]. Flat bone enchondromas such as in the pelvis, ribs, scapula, sternum, or vertebrae have been reported [2]. In contrast, chondrosarcomas secondary to enchondromas (in the context of Ollier's disease) have been reported in the axial skeleton [3]. Primary tumours of the sacroiliac joint are extremely rare and mostly consist of giant cell tumours, chondrosarcomas, and synovial villoadenomas [4]. The authors present herein an adolescent female patient with Ollier's disease of the iliac bone with sacroiliac (SI) joint involvement who posed a significant diagnostic challenge. To the authors' knowledge this is the first case of enchondromas involving the sacroiliac joint in the English-language literature. The patient and her parents were informed and gave consent that data regarding her case will be submitted for publication.

## 2. Case Presentation

**Figure 1:**
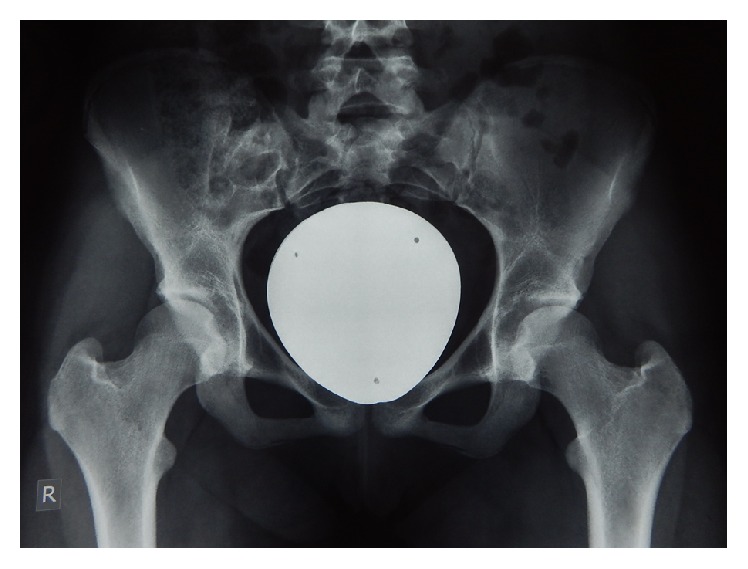
Plain radiograph of the pelvis, initially erroneously diagnosed as normal. Retrospectively, asymmetry of the SIJ is observed which was originally misdiagnosed as bowel gas.

**Figure 2:**
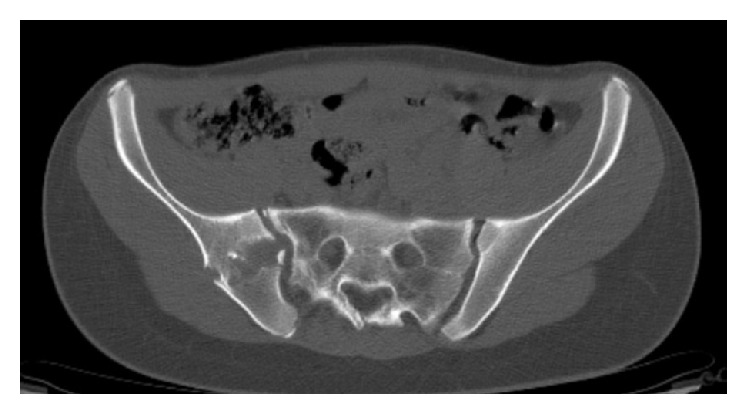
Computed tomography shows erosions of the right ilium, involving the SI joint.

**Figure 3:**
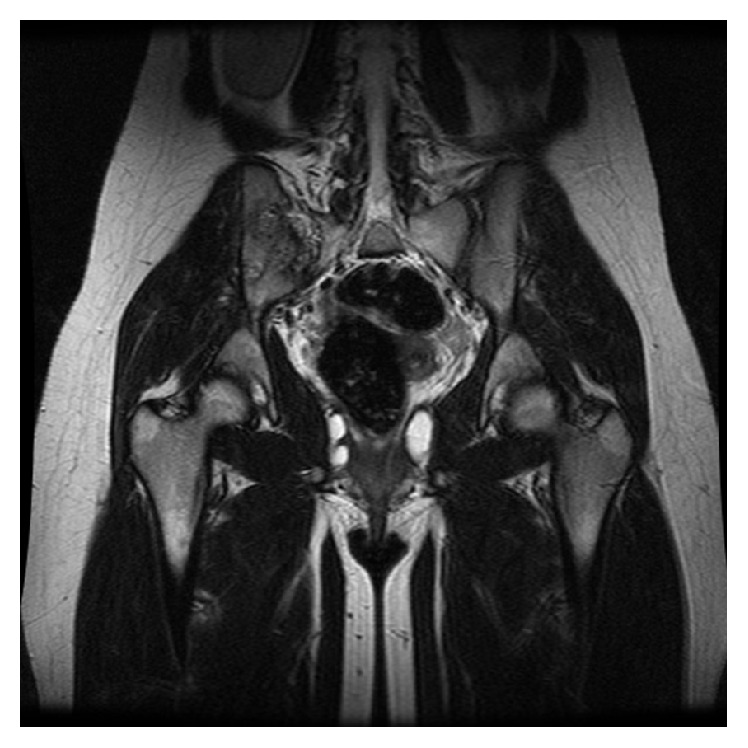
T1-sequence magnetic resonance imaging shows a heterogeneous, lobulated lytic lesion of the right ilium involving the sacroiliac joint.

**Figure 4:**
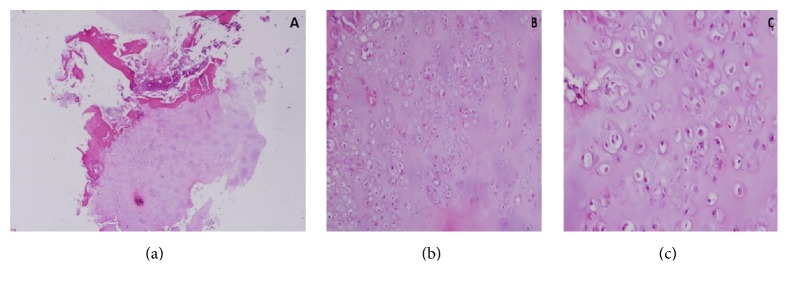
(a) Lobule of cartilaginous lesion partially surrounded by a rim of lamellar bone (H&E ×20); (b, c) relatively cellular cartilaginous lesion with mild to moderate nuclear pleomorphism (H&E ×100 and ×400).

A 16-year-old female was referred to the authors' institution complaining of low back pain (LBP) during the past 4 weeks. The patient was initially seen by a primary care physician and was managed conservatively with NSAIDs, restriction of daily activities, and physical therapy. She then visited her local orthopaedic surgeon who ordered plain X-rays of the pelvis and recommended continuation of the current treatment regimen. However, there was no improvement and thus the patient was referred to the authors' institution. Physical examination revealed point tenderness just below the right posterior superior iliac spine. Hip joint and lumbar spine motion were normal, including FABER's test (Flexion, ABduction, and External Rotation). Laboratory studies, including WBC, ESR, and CRP were noncontributory. At this point, anteroposterior radiographs of the pelvis (Figure 1) were erroneously considered as normal and therefore further imaging was requested. Computed tomography (CT) revealed significant erosions fully involving the right sacroiliac (SI) joint (Figure 2). To better characterize the lesion, magnetic resonance imaging (MRI) was ordered which showed a heterogeneous, lobulated lytic lesion of the right ilium involving the sacroiliac joint (Figure 3). At this point, a fine-needle aspiration (FNA) including cultures was nondiagnostic. A triple phase bone scan showed increased uptake in the right ilium. An open biopsy of the right SI joint was subsequently performed, which revealed several lesions in the SI joint. Histology revealed multiple foci of cartilage surrounded by a rim of lamellar bone, consistent with multiple enchondromas (Figures 4(a), 4(b), and 4(c)). The diagnosis of Ollier's disease was therefore established. The lesions underwent curettage and bone grafting. At 2-year follow-up, the patient is pain-free and has been evaluated with MRI with no evidence of recurrence.

## 3. Discussion

Low back pain increases in prevalence from 1% at 7 years of age to 17% at 12 years of age and to 53% at 15 years of age [5], simultaneously with the rapid growth spurt of adolescence [6–8]. The concept of the SI joint as a pain generator in children and adolescents is now well established [9]. Patients with SI joint pain seldom report pain above L5 [10] and most localize their pain to an area close and around the posterior superior iliac spine. The immature skeleton of the SI joint has a more flattened articular surface compared to its adult counterpart, which allows more range of motion and therefore increased risk for SI joint malalignment which may become a pain generator [11]. Other potential causes of SI joint pain include infection, trauma, stress fractures, and rarely tumours [12].

The estimated prevalence of Ollier's disease is 1 in 100,000 [13]. Ollier's disease usually manifests in first decade of life but has also been reported in early adolescence and adulthood [14]. Ollier's disease is seen twice as often in male compared to female [15]. The usual clinical manifestation of Ollier's disease includes painless asymptomatic palpable bony masses on the fingers, the toes, and the metacarpals, with a unilateral predominance [16]. Plain radiographs frequently reveal multiple lytic lesions in the affected area, with significant erosion, deep endosteal scalloping, usually greater than two-thirds of cortical thickness and no significant periosteal reaction [17]. However, the clinical presentation, including size, number, location, and evolution of lesions, as well as the requirement for surgical treatment may be highly variable [18]. Pelvic enchondromas in paediatric patients are rare and in a retrospective review of over 400 cases only a single case of solitary enchondroma of the pelvis was reported [17]. In contrast, in our case, the patient presented with atypical low back pain and point tenderness over the right SIJ. Plain radiographs were erroneously considered normal initially. In retrospect, careful scrutiny of the image shows a potential abnormality of the right SI joint. However, this is difficult to discern due to overlying bowel content. In cases of point tenderness over the SI joint, careful initial radiographic evaluation may reveal subtle but important abnormalities. Computed tomography is extremely helpful in detecting lobulated lesions, the extent of cortical erosion, endosteal scalloping, and various calcification patterns, especially in uncommon or difficult areas [19]. On MRI, the nonmineralized component of enchondromas appears as low to intermediate signal intensity lesions on T1-weighted sequences and intermediate to intense high signal lesions on T2-weighted sequences [20]. On bone scan, increased uptake can be seen with enchondromas. Intense uptake is reported with underlying pathological fracture or cortical expansion in small bones [21]. Debatable issues regarding the use of bone scans in the immature skeleton include radioprotection as well as interpretation. However, in this case, the bone scan was performed to evaluate not only the local lesions but also the potential existence of other skeletal bone lesions in a patient with probable Ollier's disease. In the current case, the results from the investigations performed were consistent with the aforementioned findings seen in Ollier's disease. Nevertheless, because the location of the lesions and the onset of symptoms were not consistent with Ollier's disease, an FNA was performed which was not diagnostic and subsequently an open excisional biopsy was undertaken.

Ollier's disease must be differentiated from a plethora of benign and malignant lesions with similar clinical presentation and radiological findings. These are, among others, low grade chondrosarcoma, nonossifying fibroma, synovial villoadenoma, simple bone cyst, aneurysmal bone cyst, fibrous dysplasia, multiple hereditary exostoses, and osteitis fibrosa cystica [17, 22–24]. Therefore, histology is critical in making the diagnosis [25]. Cartilaginous lesions partially surrounded by a rim of lamellar bone, mild to moderate nuclear pleomorphism, binucleation, and hypercellularity, are acceptable features for benign enchondromas in multiple enchondromatosis [26]. It is difficult to differentiate enchondromas from grade I chondrosarcomas until the typical bone marrow permeation with trapping of host lamellar bone on all sides is seen in the latter [27]. Depending on the size, location, onset of symptoms, and tendency to malignant transformation, the treatment varies from conservative to surgical. An often used modality is intralesional curettage which is the treatment of choice with or without bone grafting [28]. Prognosis is difficult to be assessed because of the wide clinical variability in the presentation of the disease. Frequent follow-up should be made for patients with a diagnosis of multiple enchondromas who underwent surgery [29]. In our case, because of the location, the size of the lesions, and the high potential for malignant transformation [26, 30, 31], we performed curettage with bone grafting and 2 years later the patient has no evidence of recurrence and is currently under annual surveillance.

In the English-language literature, this is the first case report of Ollier's disease of the ilium involving the SI joint, which additionally occurred in the immature skeleton of a girl. The workup of this case was challenging not only because of the unusual location, but also because of the difficulty of imaging that region with initial plain radiographs which delayed the diagnosis. Clinicians should be aware that Ollier's disease may extremely rarely occur in that location and have a very high index of suspicion in the evaluation of plain imaging of the SI joint region in cases of localized pain around the posterior superior iliac spine.
